# Sequence enrichment profiles enable target-agnostic antibody generation for a broad range of antigens

**DOI:** 10.1016/j.crmeth.2023.100475

**Published:** 2023-05-09

**Authors:** Jenny Mattsson, Anne Ljungars, Anders Carlsson, Carolin Svensson, Björn Nilsson, Mats Ohlin, Björn Frendéus

**Affiliations:** 1 BioInvent, Research, Lund, Sweden; 2 Division of Hematology and Transfusion Medicine, Department of Laboratory Medicine, Lund University, Lund, Sweden; 3 Department of Immunotechnology, Lund University, Lund, Sweden; 4 Bionamic, Lund, Sweden; 5 Section of Medical Inflammation Research, Department of Medical Biochemistry and Biophysics, Karolinska Institute, Stockholm, Sweden; 6 Broad Institute, 415 Main Street, Cambridge, MA, USA; 7 SciLifeLab Human Antibody Therapeutics, Lund University, Lund, Sweden; 8 Antibody and Vaccine Group, Centre for Cancer Immunology, School of Cancer Sciences, Faculty of Medicine, University of Southampton, Southampton, UK

**Keywords:** phenotypic antibody discovery, computational biology, therapeutic antibodies, biomarkers, specificity predictions, mathematical modeling, phage display

## Abstract

Phenotypic drug discovery (PDD) enables the target-agnostic generation of therapeutic drugs with novel mechanisms of action. However, realizing its full potential for biologics discovery requires new technologies to produce antibodies to all, *a priori* unknown, disease-associated biomolecules. We present a methodology that helps achieve this by integrating computational modeling, differential antibody display selection, and massive parallel sequencing. The method uses the law of mass action-based computational modeling to optimize antibody display selection and, by matching computationally modeled and experimentally selected sequence enrichment profiles, predict which antibody sequences encode specificity for disease-associated biomolecules. Applied to a phage display antibody library and cell-based antibody selection, ∼10^5^ antibody sequences encoding specificity for tumor cell surface receptors expressed at 10^3^–10^6^ receptors/cell were discovered. We anticipate that this approach will be broadly applicable to molecular libraries coupling genotype to phenotype and to the screening of complex antigen populations for identification of antibodies to unknown disease-associated targets.

## Highlights

• Generation of antibodies based on *in silico*-predicted antibody enrichment signatures
• Integrates computational modeling, differential antibody display selection, and NGS
• Optimizes antibody display selection by *in silico* modeling
• Generates a diverse antibody pool targeting a broad range of surface antigens

## Motivation

Phenotypic antibody discovery enables the identification of novel antibodies with the most promising functional *in vitro* or *in vivo* activity, without prior knowledge of the targeted antigen. For efficient phenotypic discovery, a large panel of antibodies against a broad repertoire of potential targets should be included in the functional testing. Current methods generate limited numbers of antibodies (10^2^–10^3^), targeting a few highly expressed antigens. This is a concern because antibodies to low-expressed antigens may have functional activity and may be relevant to biomarker discovery and therapeutic antibody development. We present a methodology that significantly enhances the number of antibodies generated (10^5^) and expands the antibody-targeted receptor expression range (antigens differentially expressed at 10^3^–10^6^ copies/cell) to include low-expressed tumor-selective antigens, enabling functional testing of a large pool of antibodies targeting a broad range of surface antigens.

## Graphical abstract

**Figure:**
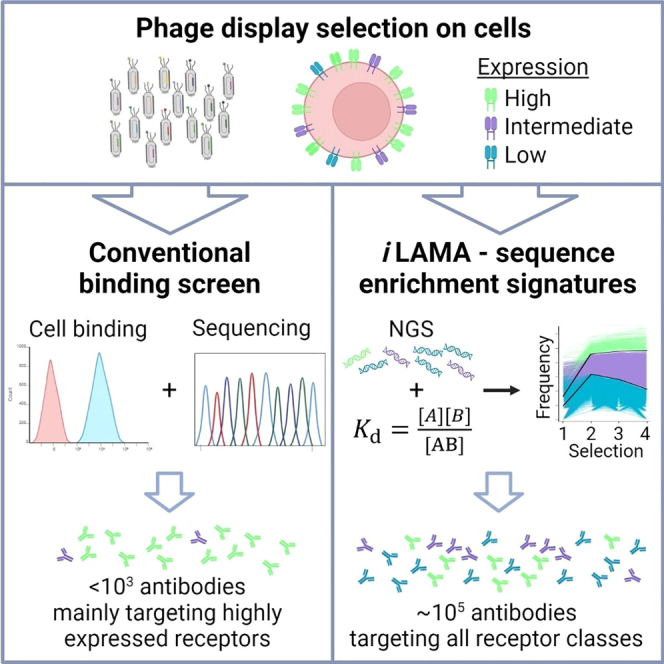

## Teaser

Phenotypic antibody discovery can identify antibodies with novel mechanisms of action, but it suffers from shortcomings in generating diverse antibody pools for functional testing. Mattsson et al. integrate computational modeling, differential antibody display selection, and massively parallel sequencing to generate diverse antibody pools to the cell surfaceome.

## Introduction

Immunotherapy with antibodies has significantly improved the survival of cancer patients^1^^,^^2^^,^^3^ and enhanced the quality of life for those with autoimmune disorders.^4^^,^^5^ Nevertheless, the lack of response and drug resistance in many patients warrant the identification of novel antibodies and therapeutically relevant targets. Phenotypic drug discovery (PDD) is a validated approach to discovering first-in-class targets and drugs.^6^ In PDD, candidate drugs from large molecular libraries are screened directly for functional activity (e.g., tumor cell death induction) without prior knowledge of targeted receptors’ identities, signaling pathways, or drugs’ mechanisms of action. Many small-molecule drugs approved by the US Food and Drug Administration (FDA) were discovered using PDD.^7^ We and others have used PDD to identify several first-in-class antibodies being trialed in clinical studies.^8^^,^^9^^,^^10^^,^^11^^,^^12^^,^^13^^,^^14^

Realizing the full potential of antibody PDD (i.e., functional screening of antibodies to all *a priori* unknown disease-associated biomolecules) will require significant improvement over existing methods. These have generated limited numbers (10^1^–10^2^) of antibodies, often specific to the most highly expressed biomolecules.^11^^,^^12^^,^^15^^,^^16^^,^^17^^,^^18^^,^^19^^,^^20^ This is a concern since antibodies to low-expressed biomolecules may have functional activity and may be relevant to biomarker discovery and therapeutic antibody development. Consequently, the main bottleneck of antibody PDD is the target-agnostic generation of antibodies to diverse disease-associated biomolecules.

Current approaches to target-agnostic antibody generation rely on a sequential process of antibody display selection followed by screening for binding to identify clones to disease-associated biomolecules (Figure S1). First, antibody pools enriched for relevant binders are generated from a genotype-linked antibody display library by applying positive selection pressure for binding to a disease-associated sample and negative selection pressure for binding to a healthy sample^21^^,^^22^^,^^23^^,^^24^^,^^25^^,^^26^^,^^27^ (Figure S1A). Individual clones from the enriched antibody pools are then screened in binding assays, either directly (Figure S1B) or following sequencing revealing the most abundant or enriched clones (Figure S1C), to identify disease-associated biomolecule-specific antibodies.^11^^,^^12^^,^^15^^,^^16^^,^^17^^,^^18^^,^^19^^,^^20^ Since target biomolecules have unknown identities and are present at unknown concentrations in disease-associated and healthy samples, the antibody selection reaction cannot be optimized to retrieve antibodies to specific target biomolecules (to be discovered). Perhaps, as a result, antibodies to low-expressed biomolecules may be present at a very low frequency (<1/10^6^) in enriched antibody pools,^28^ too low to enable their discovery by existing target-agnostic approaches.

**Figure 1:**
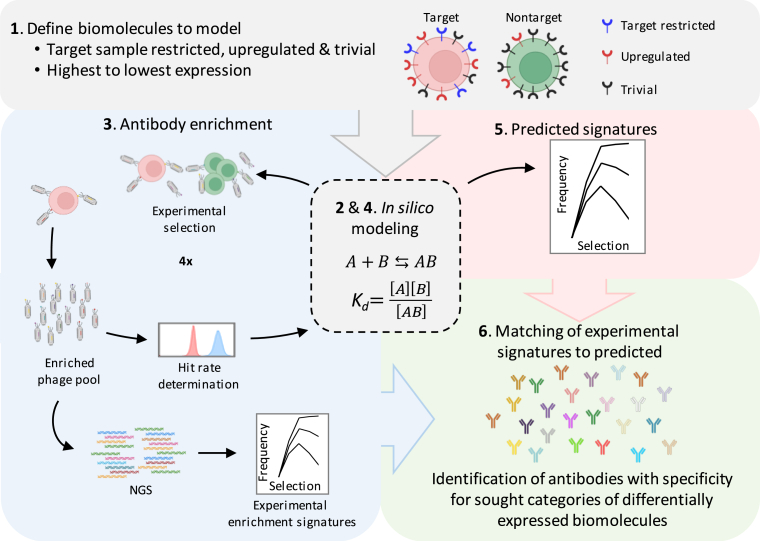
Schematic work flow of the computational modeling-based antibody discovery process *i*LaMA (1) Categories of (hypothetical) biomolecules, e.g., surface receptors, are defined by their absolute and relative expression levels on target and nontarget cells. Biomolecule expression levels ranging from no expression to highest estimated expression on target and nontarget samples are included and used to model selection. (2) The law of mass action-based computational modeling is performed to optimize selection reaction parameters (e.g., numbers of target and nontarget cells) which are then (3) used in experimental selection to enrich displayed antibodies to sought categories of differentially expressed biomolecules. Enriched antibody pools are analyzed by massively parallel next generation sequencing (NGS) to provide experimental antibody enrichment signatures. The fraction of antibodies that has been enriched in a target biomolecule-dependent manner (the hit rate) is determined. (4 and 5) The law of mass action-based *in silico* modeling is used to generate predicted antibody enrichment signatures for antibodies to the different categories of biomolecules. (6) Finally, experimental and predicted enrichment signatures are matched to identify antibody sequences encoding specificity to sought categories of differentially expressed biomolecules. See also Figure S1.

However, antibody binding to biomolecules is known to occur in an affinity (K_d_)-driven and target and antibody concentration-dependent manner, which can be described by the universal law of mass action (LaMA). We hypothesized that a computational biology approach where enrichment of antibodies is modeled using the LaMA, according to antibody specificity for biomolecules expressed at different levels throughout disease-associated and healthy sample biomolecule expression ranges, could help optimize the selection and identification of medically relevant antibodies. Here, we describe a rational discovery approach that overcomes the quantitative and qualitative limitations of the current state-of-the-art target-agnostic discovery methods. Integrating the LaMA-based computational modeling, experimental antibody selection, and massively parallel sequencing, *i*LaMA optimizes antibody display selection and predicts which selected antibody sequences encode specificity to disease-associated biomolecules (Figure 1). Our approach enables the rational identification of tens of thousands of unique antibody sequences encoding specificity for diverse *a priori* unknown disease-associated biomolecules.

## Results

### *i*LaMA accurately predicts antibody enrichment according to targeted receptor specificity

To overcome the quantitative and qualitative limitations of current approaches to generate antibodies for biologic PDD, we developed *i*LaMA, a computational biology approach that integrates the law of mass action-based mathematical modeling and antibody display selection to identify medically relevant antibodies. The key steps of this methodology include optimizing and performing antibody display selection, modeling biomolecule-specific antibody enrichment, and matching computationally predicted and experimentally obtained antibody sequence enrichment profiles to identify antibody clones encoding specificity to sought types of disease-associated biomolecules, as summarized in Figure 1.

We applied this methodology to screen the large (>10^10^ members) naive human phage display library n-CoDeR^26^ for antibodies to cell surface receptors differentially expressed between two cell types: DU145 prostate cancer (target) cells and Jurkat (nontarget) T cells. In the first step, we defined categories of differentially expressed surface receptors on these cells spanning an estimated expression range of 10^3^–10^6^ receptors per cell (STAR Methods). We used *i*LaMA to identify the number of input cells needed for the enrichment of high-affinity (K_d_ ≤ 10 nM) antibodies to target cell receptors >5-fold upregulated on target cells compared with nontarget cells and depletion of antibodies to receptors <5-fold upregulated on target cells, expressed throughout this range (10^3^–10^6^ receptors/cell). In our model, the number of antibodies retrieved following selection is a function of the number of biomolecules (receptors) on the target and nontarget cells and the number of target and nontarget cells used in the selection reaction, with the formula

$$rA_{T}=\left(\left(\frac{A+B+K_{d}N_{A}V}{2}-\sqrt{\frac{{\left(A+B+K_{d}N_{A}V\right)}^{2}}{4}-AB}\right)\times \left(\frac{B_{T}C_{T}}{B_{T}C_{T}+B_{N}C_{N}}\right)\right)\times E\times Y$$

where rA_T_ is the number of retrieved antibodies on the target cells, A is the total number of antibodies, B is the total number of biomolecules on target and nontarget cells, K_d_ is the antibody affinity (M), N_A_ is Avogadro’s constant (6,022 × 10^23^ molecules/mole), V is the selection reaction volume (dm^3^), C_T_ and C_N_ are the numbers of target and nontarget cells, B_T_ and B_N_ are the number of biomolecules on C_T_ and C_N_, Y is the fraction of recovered target cells following selection, and E is the fraction of antibodies eluted from C_T_.

*i*LaMA indicated that 1 × 10^7^ target cells were needed in the selections to allow enrichment of antibodies to low-expressed receptors and that a 1,000-fold excess of nontarget cells was needed to efficiently remove antibodies to receptors <5-fold upregulated on target cells compared with nontarget cells while enriching antibodies to receptors >5-fold upregulated. *i*LaMA also predicted that the enrichment of antibodies to >5-fold upregulated receptors would markedly differ between selections with and without competition (Figure S2). The latter indicated that comparative antibody enrichment profiles generated from the two selection types could be used to discriminate antibody sequences encoding specificity for (target cell) restricted and upregulated receptors.

We performed experimental selection with and without competition using the calculated cell numbers and generated sequence enrichment profiles of individual antibody clones by massively parallel sequencing of the selected antibody pools. We used four rounds of selection aiming to generate robust and different enrichment signatures for antibody sequences encoding specificity for different types of receptors. We used *i*LaMA to generate predicted enrichment signatures for antibodies to biomolecules with defined expression levels on target and nontarget cells using a K_d_ of 10 nM (corresponding to the median affinities of antibodies to diverse cell surface receptors isolated from herein using the n-CoDeR antibody library^10^^,^^29^^,^^30^) and the formula

$$FA_{T}=\frac{rA_{T}}{\left(\sum_{1}^{n}rA_{T}\right)}\;x\;HR$$

where FA_T_ and rA_T_ are the frequencies and numbers of recovered antibodies specific for a given type of target cell biomolecule and HR is the hit rate (i.e., the fraction of phage antibodies that has been enriched in an antibody and target cell receptor-dependent manner in each selection round as determined by flow cytometry). The HR was incorporated since the enrichment of non-specific (antibody-target cell receptor non-specific) phage antibodies, which typically dominate during early selection rounds^18^^,^^31^ (Table S1), appears stochastic and cannot be modeled using the LaMA. By experimentally determining the fraction of phage antibodies that have been enriched in an antibody-antigen-specific manner and then selectively modeling this, *i*LaMA considers the presence of the non-specific antibody fraction yet enables the focused discovery of antibody sequences encoding specificity for differentially expressed target cell receptors, according to their predicted enrichment profiles.

**Figure 2:**
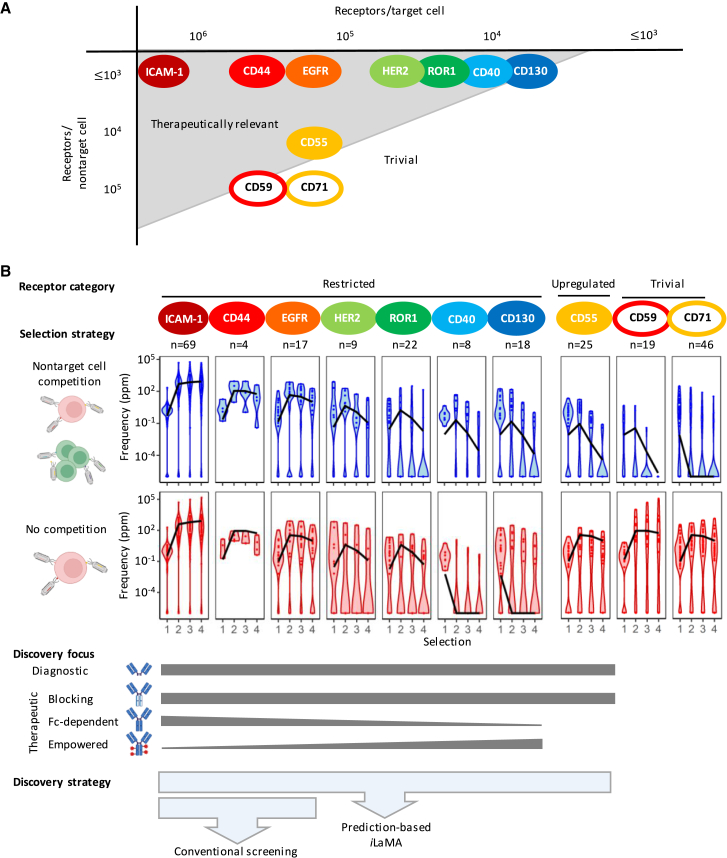
*i*LaMA models antibody enrichment according to targeted receptor’s absolute and relative expression levels (A) Overview of 10 reference receptors used to evaluate prediction-based discovery, showing the number of receptors on target and nontarget cells. The selection conditions used in this study were optimized for the discovery of antibodies targeting receptors in the gray area. (B) Predicted signatures and outcomes. Black lines show *in silico*-modeled signatures for antibodies targeting the reference receptors. Violin plots show experimental enrichment of antibodies targeting these receptors in selections with nontarget cell competition (blue) and without nontarget cell competition (red). Gray areas indicate the range of diagnostically and therapeutically relevant receptors and the therapeutic applicability of antibodies targeting these receptors. Blue areas indicate whether antibodies targeting the receptors can be found with conventional screening technology and/or prediction-based discovery. See also Figures S2, S3, and Table S2.

To evaluate how well the predicted enrichment signatures matched experimental signatures of antibody clones selected from n-CoDeR, we used reference antibody sequences encoding specificity to known receptors with a determined expression on target and nontarget cells and comprising both target-cell-restricted and upregulated receptors (Figure 2A and Table S2). The numbers of molecules per target cell of restricted receptors spanned 10^3^ (CD130), 10^4^ (CD40, ROR1, HER2), 10^5^ (CD44, EGFR), and up to 10^6^ (ICAM-1) receptors per target cell, with no detectable expression on nontarget cells. Upregulated receptors CD55, CD59, and CD71 were similarly expressed on target cells (2–4 × 10^5^ receptors/cell) but were >5-fold (CD55, 10-fold) or <5-fold (CD59 4-fold, and CD71 2-fold) upregulated on target cells compared with nontarget cells (Figure 2A; Table S2). We used *i*LaMA to predict enrichment signatures of model antibodies (K_d_ = 10 nM) to these receptors and compared these signatures with experimental enrichment signatures of reference antibodies to the same receptors. A strong correlation between *in silico-*predicted and experimentally determined frequencies was observed for antibodies to all reference receptors in both selections with and without nontarget cell competition. Notably, as predicted by the computational modeling, the signatures for antibodies to low-expressed target-cell-restricted receptors (CD40 and CD130) and upregulated receptors (CD55) were very similar in selections with nontarget cell competition but differed markedly in selections without competition (Figure 2B).

Since selection of antibodies from highly diversified libraries will generate receptor-specific antibodies of varying affinity, we additionally modeled antibody enrichment according to 10-fold higher (1 nM) or 10-fold lower (100 nM) affinities. While enrichment signatures were overall similar, the modeled antibody frequencies increased with higher affinity and decreased with lower affinity, mainly affecting restricted receptors (Figure S3A). To assess how possible errors in receptor quantification might affect antibody enrichment signatures, we additionally modeled enrichment according to 2-fold higher or 2-fold lower numbers than experimentally determined. This mainly affected signatures of antibodies to upregulated receptors (CD71, CD59, CD55; Figure S3B).

In summary, *in silico*-modeled enrichment of target receptor-specific antibodies closely mirrored experimental antibody enrichment, which was driven by the absolute and relative expression levels of targeted receptors on target and nontarget cells and by the presence or absence of competition selection.

### *i*LaMA indicates tens of thousands of antibodies to differentially expressed receptors in selected antibody pools

**Figure 3:**
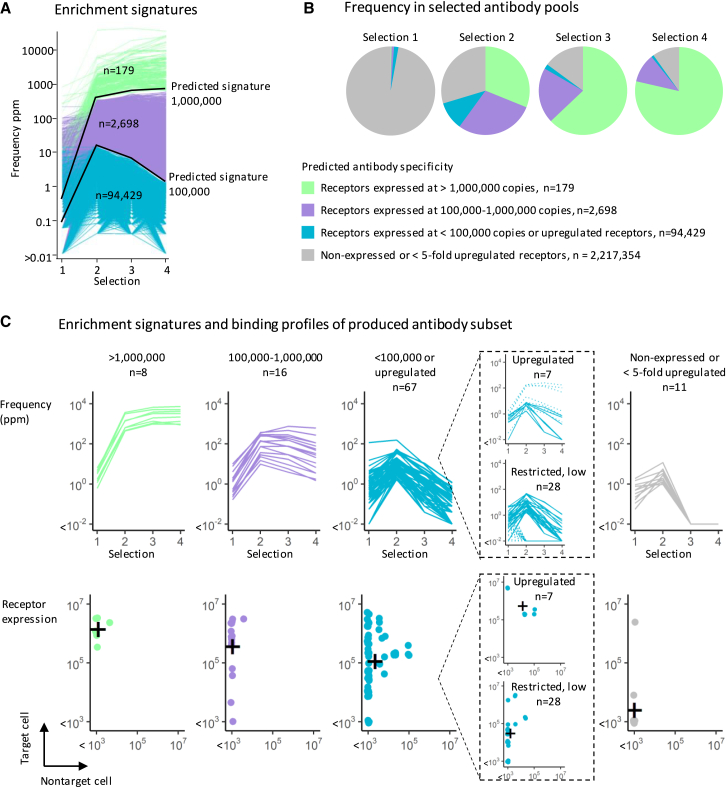
*i*LAMA-predicted sequence enrichment profiles inform antibody specificity, enabling focused discovery of antibodies to differently expressed *a priori* unknown tumor cell-associated receptors (A) Enrichment signatures showing antibody sequence frequencies after selections 1 to 4. Black lines show *in silico*-predicted signatures for antibodies targeting receptors expressed at 1 million and 100,000 copies/target cell with no expression on nontarget cells. By matching experimental signatures to predicted, experimentally enriched antibodies were classified into three groups predicted to bind target receptors expressed at > 1 million copies/cell (green, n = 179), 100,000–1 million copies/cell (purple, n = 2,698), or <100,000 copies/cell or upregulated receptors (blue, n = 94,429). (B) The frequency of experimentally enriched antibodies to different receptor categories after selections 1–4. Gray shows antibodies predicted to not bind target cells or to bind receptors expressed at similar levels on target and nontarget cells. (C) A subset of antibodies from each enrichment signature group (top panel) was produced and tested for binding to target and nontarget cells (bottom panel). The cross represents the geometric mean of receptor expression on target and nontarget cells. Inset: antibodies in the <100,000 or upregulated group further classified as binding upregulated or restricted low-expressed receptors based on comparative analyses of selections with nontarget cell competition (solid lines) and selections without nontarget cell competition (dotted lines). See also Figure S4.

Our results demonstrated that *i*LaMA accurately modeled the enrichment of antibodies to receptors expressed over a wide dynamic range. This indicated its potential utility as a discovery tool to identify antibodies of distinct therapeutic and diagnostic value without *a priori* knowledge of the antibody target identities. The method’s importance for such discovery would, however, be determined by the number of antibodies the technology could generate. To assess this, we queried the selected antibody pools for antibody sequences with enrichment profiles matching *in silico*-predicted signatures of antibodies specific to receptors with expression profiles indicating therapeutic or diagnostic relevance. Diagnostic antibodies and therapeutic antibodies that rely purely on blockade of ligand-receptor signaling (e.g., anti-interleukin [IL]-6R^32^) could be specific to receptors expressed over a wide dynamic range. In contrast, Fc-dependent or empowered therapeutic antibodies, mediating, e.g., antibody-dependent cellular cytotoxicity (ADCC) or chimeric antigen receptor (CAR)-T cell specificity, to low-expressed tumor-restricted antigens can have significant therapeutic potential but require low or no receptor expression on critical normal cells (Figure 2B). Thus, individual antibody sequences with enrichment signatures matching those predicted to represent specificity for restricted receptors expressed at (1) >10^6^ molecules/target cell, (2) 10^5^–10^6^ molecules/target cell, or (3) <10^5^ molecules/target cell *or* being >5-fold upregulated on target compared with nontarget cells were quantified. Analysis of the complete sequence dataset indicated the presence of tens of thousands of unique antibody clones to the above three receptor categories (Figure 3A). The signature-guided analyses further indicated that sequences encoding antibodies specific to the most highly expressed receptors dominated binder pools (10^2^–10^5^ ppm/sequence) following two or more selection rounds. Conversely, antibodies to lower-expressed restricted receptors or upregulated receptors were rare (10^−2^–10 ppm/sequence). Interestingly, and in contrast to their low frequency, the *number* of antibody clones with indicated specificity for upregulated or target-restricted receptors with low expression exceeded 10^4^ by the same analyses (Figures 3A and 3B).

As discussed above, upregulated and target-restricted receptors exhibit therapeutic potential as Fc-dependent and empowered antibodies, respectively. Since enrichment signatures of antibodies to these two types of receptors differed between selections with or without competition (Figures 2B and S4A), we performed comparative analysis of the total 94,429 sequences indicated by enrichment signatures to encode antibodies to low-expressed or upregulated receptors. This allowed a fraction of the antibodies to be classified into either category. Accordingly, 13,709 antibodies were indicated to be specific for low-expressed restricted receptors (increasing frequency in selections with competition compared with without), and 753 antibodies were indicated to be specific for upregulated receptors (decreasing frequency in the presence of competition) (Figure S4B). The remaining unclassified antibodies were less clearly affected by nontarget cell competition.

### Predicted sequence enrichment profiles inform antibody specificity and enable the discovery of antibodies to *a priori* unknown tumor-cell-associated receptors

Our collective data thus indicated that prediction-guided selections generated a highly diversified pool of antibodies to *a priori* unknown differentially expressed surface receptors covering a broad expression range. Furthermore, enrichment signatures identified unparalleled numbers (∼10^5^ compared with <10^3^) of antibodies to therapeutically and diagnostically relevant receptors. To understand its relevance for such antibody discovery, a subset of antibody sequences (n = 102) indicated by enrichment signatures to encode antibodies specific for restricted receptors expressed at (1) >10^6^ molecules/cell (n = 8), (2) 10^5^–10^6^ molecules/cell (n = 16), (3) <10^5^ molecules/cell *or* target cell upregulated receptors (n = 67), or (4) antibodies to similarly expressed receptors (<5-fold difference) *or* nonreceptor specific antibodies (n = 11) were selected for production in full-length immunoglobulin (Ig) G format and quantitation of antibody-targeted receptor expression (Figure 3C). This analysis showed that 93% (85 out of 91) of the antibodies in groups 1–3 were specific to differentially expressed receptors on target cells. Equally essential and demonstrating the power of this prediction-guided discovery approach, 9 out of 11 (82%) of the antibodies in group 4 did not bind target cells. These antibody clones showed apparent enrichment between selection rounds 1 and 2 yet were indicated by enrichment signatures to lack specificity for medically relevant receptors. With existing target-agnostic methods, which do not use *i*LAMA predictive enrichment signatures but rather pick clones randomly or based on the greatest enrichment during selection, these medically trivial antibodies would have been selected for time- and resource-consuming production and downstream screening at the cost of medically relevant clones (e.g., antibodies specific to lower expressed biomolecules).

The above results demonstrated that *i*LaMA-predicted enrichment signatures could be used with high accuracy to identify which antibody sequences encode specificity to medically relevant or trivial receptors. Consistent with enrichment signatures informing antibody specificity, predicted and experimentally determined receptor expression correlated well (Figure 3C). Antibodies in group 1 were predicted to bind receptors with >10^6^ copies/cell, and the experimentally determined receptor expression was 1.4 × 10^6^ (7.3 × 10^5^ to 2.6 × 10^6^) (geometric mean [95% confidence interval]). Group 2 was predicted to bind receptors with expression levels between 10^5^ and 10^6^, and the experimentally determined number was 3.6 × 10^5^ (1.0 × 10^5^ to 1.3 × 10^6^). Finally, group 3 was predicted to bind restricted receptors with expression levels <10^5^ *or* upregulated receptors. To help separate these different categories of antibodies, we performed comparative analyses of group III enrichment signatures from selections with or without nontarget cell competition. The studies revealed sequences that showed either decreasing (7 out of 67) or increasing (28 out of 67) frequency with nontarget cell competition compared with without nontarget cell competition (Figure 3C, inset). Consistent with decreasing frequency indicating antibody specificity for upregulated receptors, the experimentally determined receptor number on target cells and nontarget cells for antibodies with decreasing frequency was 5.4 × 10^5^ (1.4 × 10^5^ to 2.2 × 10^6^) and 1.3 × 10^4^ (2.3 × 10^3^ to 7.9 × 10^4^), respectively. Conversely, and consistent with an increasing frequency indicating antibody specificity for restricted low-expressed receptors, the experimentally determined receptor number on target and nontarget cells for antibodies with increasing frequency was 3.0 × 10^4^ (1.3 × 10^4^ to 7.1 × 10^4^) and undeterminable, the latter since 22 out of 28 antibodies bound receptors with nontarget cell expression below the detection limit (Figure 3C, inset).

While, overall, antibody specificity could be predicted by the enrichment signatures, the specificity of some individual antibodies deviated from predictions (Figure 3C). For example, only group 1 (green, left panel) was predicted to contain antibodies specific for receptors expressed at >1 million copies/target cell with no expression on nontarget cells. However, all groups contained a fraction of antibodies with this expression profile. Since our modeling data indicated differential enrichment of antibodies with varying affinity to the same receptor (or receptors with the same expression level) and naive antibody libraries contain antibodies of varying affinity, we next analyzed how antibody affinity affected antibody enrichment signatures. Antibodies with similar determined epitopes (i.e., similar receptor expression levels on target cells) were divided in two groups according to their >3nM or <3nM half-maximal effective concentration (EC_50_) values for binding to endogenously expressed receptors and their enrichment during selection was compared. Consistent with our modeling (Figure S3A), for both monitored target expression levels (1–4 million copies per cell, n = 22, and 300.000–1 million copies per cell, n = 10) we found the frequencies of antibodies with an EC_50_ value <3 nM were higher than the frequencies of antibodies with an EC_50_ value >3 nM. For antibodies to receptors with 1–4 million copies per cell, this difference was statistically significant (p < 0.05) after selections 2, 3, and 4 (Figure S4C).

**Figure 4:**
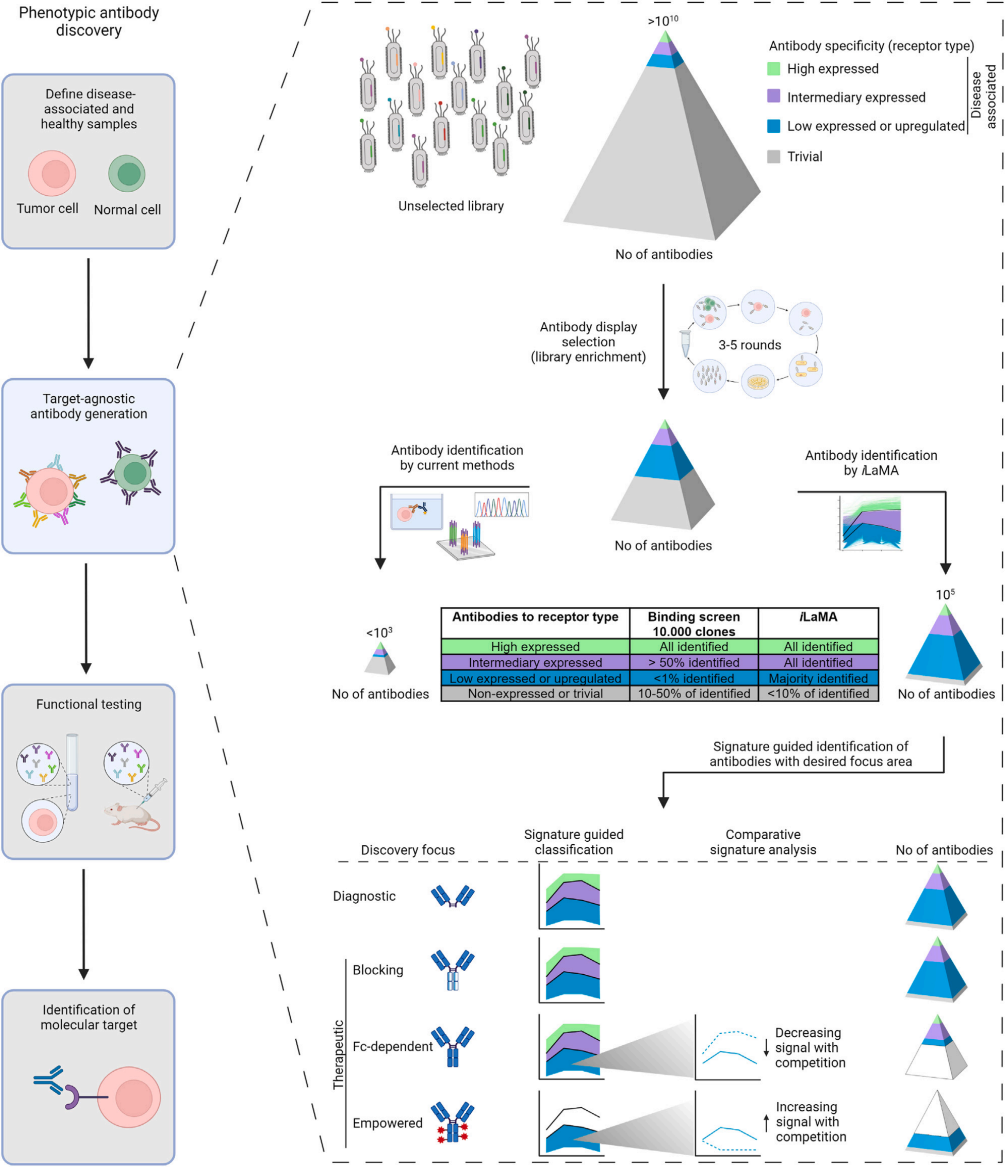
*i*LaMA target-agnostic discovery of antibodies to diverse disease-associated biomolecules *i*LaMA enhances the quantitative output of antibodies from target-agnostic discovery by orders of magnitude (10^5^ compared with <10^3^). This computational prediction-based approach further allows focused discovery of antibodies by intended therapeutic or diagnostic application(s). For example, preferential identification of antibody clones with potential in therapeutic development as naked blocking IgGs, or empowered antibody-drug conjugates (ADCs), is enabled through comparative signature analysis and picking of clones with indicated specificity for any disease-associated biomolecules, or low-expressed biomolecules expressed only in disease-associated sample, tissues or cells, respectively. *i*LaMA enables rational PDD of antibodies according to their indicated therapeutic potential.

In summary, prediction-guided discovery enabled the identification of unprecedented numbers of antibodies binding to differentially expressed surface receptors of distinct potential therapeutic or diagnostic value (Figure 4).

## Discussion

Here, we describe a target-agnostic antibody generation methodology that enables the rational identification of antibodies to *a priori* unknown differentially expressed cell surface receptors. Through computational prediction of antibody enrichment, this methodology enabled the discovery of antibodies to biomolecules expressed throughout therapeutic and diagnostic relevant ranges and identified orders of magnitude greater numbers of antibodies than existing technologies (Figure 4). As such, our computational science-based discovery overcomes several limitations of the current state-of-the-art selection and screening methodology and has the potential to transform biological drug, target, and biomarker discovery.

Predictions will be beneficial to drug developers interested in biologics PDD (for a recent review on PDD, see Moffat et al.^33^). In PDD, small molecules or antibodies from large molecular libraries are screened for functional activity (e.g., inhibition of proinflammatory cell cytokine release or induction of tumor cell death) without prior knowledge of their molecular targets. Consequently, PDD enables the discovery of the most functional molecules and antibodies across multiple receptors, epitopes, and disease-associated pathways. PDD is a well-validated strategy for first-in-class small-molecule drug discovery,^6^^,^^34^^,^^35^ and we and others have used PDD to identify first-in-class antibodies to, e.g., CD52,^8^ ICAM-1,^9^ CD32b,^10^^,^^11^ and TNFR2,^12^ several of which are currently in clinical development.^36^^,^^37^^,^^38^^,^^39^^,^^40^ By combining prediction-based antibody discovery with appropriate functional screening^11^^,^^41^ and our recently described high-throughput CRISPR-based method for target deconvolution,^42^ biologic PDD can be taken to the next level, directly on par with small-molecule PDD.

A key feature of prediction-based discovery relevant to diagnostic and therapeutic applications is its ability to identify antibodies to poorly expressed disease-associated molecules. Antibodies to low-expressed tumor-restricted antigens and rare disease-associated configurational epitopes may have significant therapeutic potential when developed as empowered biologics such as CAR-T cells or ADCs. Our observations that antibodies to low-expressed and upregulated receptors are present at a shallow frequency (one or fewer clones per million) in selected pools are consistent with the observed shortcoming of existing methods to generate such antibodies. In the absence of integrated computational modeling and informed enrichment signatures in data generated by massively parallel sequencing, robust identification of these specificities would require production and labor-intensive cell-based screening of millions of antibody clones (Figures 3 and 4).

Antibodies to low-expressed receptors are also of value in diagnostics. While liquid biopsies can be easily sampled, they typically contain biomarkers in very low concentrations. Technologies that help identify and quantitate rare disease-associated biomarkers are therefore instrumental to pursuing an earlier diagnosis and personalized medicine.

In this study, we used our computational modeling approach to antibody discovery to identify antibodies that discriminate one cell type from another. Accordingly, the methodology can be used to isolate antibodies to overexpressed targets on, e.g., primary tumor cells and tumor-infiltrating lymphocytes compared with healthy samples or resistant cancer cells compared with drug-sensitive cancer cells. However, the method is also broadly applicable to other complex antigen systems (e.g., blood, urine, cerebrospinal fluid,^43^ tissue,^44^ bacteria^45^ or viruses^46^) relevant to diverse inflammatory, immunological, neurological, infectious diseases, and cancer. A currently highly relevant application is identifying antibodies to crucial virulence factors (e.g., adhesive glycoproteins of pandemic microorganisms^47^) and their receptors on host cells. Identifying such antibodies and their associated molecular targets can generate both passive (antibody-based) and active (vaccine) immunotherapies to help treat and prevent drug-resistant infections.

A final key advantage of our prediction-based discovery approach relative to other discovery methodologies (e.g., gene expression-based or proteomics-based approaches) is the parallel discovery of target molecules and of candidate therapeutic or diagnostic human antibodies against these targets, which may be of a composite or processed nature (e.g., oxidized low-density lipoprotein^30^^,^^48^^,^^49^) and may include disease-associated epitopes and configurations.

Regarding limitations, although antibody enrichment signatures correlate well with receptor expression levels on target and nontarget cells, this is not the case for all individual antibody clones (Figure 3C). Antibody enrichment is affected by several factors that are incompletely considered or not considered by the model. These include varying affinities; individual antibody affinities may deviate significantly from the K_d_ value used to model antibody enrichment according to targeted molecules’ expression levels (as shown in Figure S3), accessibility of targeted epitope, and phage amplification in bacteria. The library used in this study was generated using a single framework. Hence, only the complementarity-determining regions vary between the antibodies. This minimizes sequence composition differences and associated growth bias during phage amplification. However, such differences may have a bigger impact when the method is applied to libraries with different frameworks. Despite these limitations, predictions enabled the preferential discovery of antibodies with the desired specificity.

Another limitation is the cost of antibody production. Synthesizing thousands of antibody genes discovered by the method is still associated with a considerable cost. By focusing on a particular class of antibodies, as informed by enrichment signatures, the number of antibodies to test can be dramatically reduced (Figure 4). Additionally, as new, more efficient, cost-effective gene synthesis methods are developed, more antibodies can be tested.

In this work, we used the Illumina platform to sequence the antibody pools. While this allows high-accuracy deep sequencing of CDRH3 (here, a median of 25 million usable reads/sample), the read length is too short to cover the entire antibody sequence. Recently, the accuracy and yield of long-read sequencing have approached those of short-read sequencing.^50^ Implementation of long-read sequencing would make it possible to distinguish different antibodies with the same CDRH3 in the enrichment signatures and eliminate the need for a second sequencing strategy to generate the full antibody sequence for gene synthesis of the selected antibody clones.

In conclusion, and with reference to PDD, we expect prediction-based discovery to shift the current bottleneck from identifying many antibodies to all differentially expressed, disease-associated biomolecules to developing efficient antibody production and high-throughput clinically predictive functional assays, which allow for the screening of thousands of clones. We recently described functional assays that allow for high-throughput screening of antibody-mediated apoptosis and ADCC using primary patient cancer cells and immune effector cell lines.^11^ With current instrumentation, these assays could easily be adapted for orders-of-magnitude-greater throughput and use with lower numbers of primary human cells. Encouraging advances in high-throughput antibody characterization will help prioritize clones with unique or highly desired characteristics (e.g., high affinity^18^ or different MoA^41^) for testing with primary patient material in the most clinically relevant assays. Such advancements will hopefully lead the way toward a broader range of even more effective therapeutics for patients.

### Limitations of the study

The method described here enables the target-agnostic generation of a large panel of antibodies to a broad range of differentially expressed antigens. In this study, we used the phage display library n-CoDeR to generate antibodies targeting the cancer cell line DU145. While the method should be applicable to other libraries, some parameters, such as mean affinity and the number of copies of individual antibodies in the unselected library, will have to be adjusted. Further, while additional sources of complex targets can be screened, it is important that the target and nontarget populations stay consistent throughout the selections. If liquid samples such as blood are used, the method used to capture soluble components must be reproducible. The inclusion of reference targets with known expressions in target and nontarget samples can be used to ensure proper modeling. While the method helps design display selections and guide the discovery of antibodies with the desired specificity, enrichment signatures of individual antibody clones may deviate from those modeled due to, e.g., varying affinity, target epitope immunogenicity, and phage amplification in bacteria.

## STAR★Methods

### Key resources table

**Table:** 

REAGENT or RESOURCE	SOURCE	IDENTIFIER
**Antibodies**		

APC Mouse anti-Human CD54	BD Biosciences	Cat# 559771; RRID:AB_398667
APC Mouse anti-Human CD44	BD Biosciences	Cat# 559942; RRID:AB_398683
PE Mouse anti-Human EGF Receptor	BD Biosciences	Cat# 555997; RRID:AB_396281
PE Mouse anti-Human HER-2/Neu	BD Biosciences	Cat# 340552; RRID:AB_400055
PE Mouse anti-Human ROR1	BD Biosciences	Cat# 564474; RRID:AB_2738822
PE Mouse anti-Human CD40	BD Biosciences	Cat# 555589; RRID:AB_395964
PE Mouse anti-Human CD130	BD Biosciences	Cat# 555757; RRID:AB_396098
PE Mouse anti-Human CD55	BD Biosciences	Cat# 561901; RRID:AB_10893598
PE CD59 Antibody, anti-human, REAfinity™	Miltenyi	Cat# 130-120-048; RRID:AB_2751973
APC Mouse anti-Human CD71	BD Biosciences	Cat# 551374; RRID:AB_398500
His Tag Alexa Fluor® 647-conjugated Antibody	R&D Systems	Cat# IC0501R
Mouse anti-FLAG M2, AP-conjugated	Sigma-Aldrich	Cat# A9469; RRID:AB_439699
Goat anti-human-Fc, APC conjugated	Jackson Immunoresearch	Cat# 109-136-098; RRID:AB_2337693

**Bacterial and virus strains**		

*E*.*coli* HB101F′	In house produced	N/A
*E*.*coli* Top10	Thermo Fisher Scientific	Cat# C404010
R408 Helper phage	Agilent Technologies	Cat# 200252

**Chemicals, peptides, and recombinant proteins**		

ICAM-1	R&D Systems	Cat# 720-IC
CD44	Sino Biological	Cat# 12211-H08H
EGFR	Sino Biological	Cat# 10001-H08H
HER2	Sino Biological	Cat# 10004-H08H
ROR1	Sino Biological	Cat# 13968-H08H
CD40	In house produced	N/A
CD130	Sino Biological	Cat# 10974-HCCH
CD55	Sino Biological	Cat# 10101-H08H
CD59	Sino Biological	Cat# 12474-H08H
CD71	Sino Biological	Cat# 11020-H07H

**Experimental models: Cell lines**		

Human: DU 145	ATCC	Cat# HTB-81
Human: Jurkat, Clone E6-1	ATCC	Cat# TIB-152
Human: HEK293 EBNA	ATCC	Cat# CRL-10852

**Oligonucleotides**		

Forward MiSeq1: 5′-AATGATACGGCGACCA CCG AGATCTACACTCTTTCCCTACACGACGCTCTTCC GATCTttccctgagactctcctgtgcagcctctggattcacctt-3′ Upper case - sequencing adaptors, lower case - scFv primers	This paper	N/A
Forward MiSeq2, NextSeq: 5′-AATGATACGG CGA CCACCGAGATCTACACTCTTTCCCTACACGACGC TCTTCCGATCTtagagccgaggacactgccgtgtattactgt-3′	This paper	N/A
Reverse MiSeq1, NextSeq: 5′-CAAGCAGAAG ACGGCATACGAGAT – 10nt Index – GTGACTGGAGTTCAGACGTGTGCTCTTCCG ATCTcgctgctcacggtgaccagtgtaccttggcccca-3′	This paper	N/A
Reverse MiSeq2: 5′-CAAGCAGAA GACGGCATACGAGAT – 10nt Index – GTGACTGGAGTTCAGACGTGTGCTCT TCCGATCTgtcagcttggttcctccgccgaa-3′	This paper	N/A

**Software and algorithms**		

FlowJo v10.7.2	FlowJo, LLC	https://www.flowjo.com/solutions/flowjo
GraphPad Prism 9.5.1	GraphPad	https://www.graphpad.com

### Resource availability

#### Lead contact

Further information and requests for resources and reagents should be directed to and will be fulfilled by the lead contact, Björn Frendéus (bjorn.frendeus@bioinvent.com).

#### Materials availability

Antibodies generated in this study will be made available on request under a completed materials transfer agreement.

### Experimental model and subject details

#### Tissue culture

Human prostate carcinoma cell line DU145 (ATCC) was cultured in MEM (Gibco) containing 10% fetal bovine serum (FBS, Gibco), 1mM Sodium Pyruvate (Gibco) and 1x MEM Non-Essential Amino Acids Solution (Gibco). Acute T cell leukemia cell line Jurkat (clone E6-1, ATCC) was cultured in RPMI-1640 with GlutaMAX (Gibco) containing 10% FBS and 1mM Sodium Pyruvate. The cells were grown at 37°C in a humidified atmosphere and 5% CO_2_.

In-house suspension adapted HEK 293 EBNA (ATCC) was cultured in Freestyle293 medium (Thermo Fisher Scientific) supplemented with 10% Pluronic F-68 (Thermo Fisher Scientific) at +37°C, 8% CO2, 300 rpm in a humidified atmosphere.

### Method details

#### Derivation of iLaMA equations

To optimize the selection conditions, the recovery of antibodies to different categories of target biomolecules was modeled *in silico*. Following experimental selections, the actual experimental parameters were inserted into the equations, and predicted enrichment signatures were modeled.

According to the law of mass action, the interaction between an antibody (A), its target biomolecule (B), and their complex (AB) is given by the equilibrium interaction

A+B⇌AB

with the equilibrium dissociation constant or affinity

$$K_{d}=\frac{\left[A\right]\left[B\right]}{\left[AB\right]}$$

The equilibrium interaction between (A) and (B) may be described as

BoundA(bA)⇌freeA(fA)+freeB(fB)

with

(Equation 1) $$Kd=\left[fA\right]\times \frac{\left[fB\right]}{\left[bA\right]}$$

The total A or B ([*A*] or [*B*]) is the sum of free and bound A or B, i.e.,

[A]=[fA]+[bA],and[B]=[fB]+[bA]

Therefore, in Equation 1, replacing [fA] by [*A*]-[bA] and [fB] by [*B*]-[bA] gives

(Equation 2) $$K_{d}=\left(\left[A\right]-\left[bA\right]\right)\times \frac{\left[B\right]-\left[bA\right]}{\left[bA\right]}$$

which is rearranged to form

$$\left(K_{d}\left[bA\right]\right)=\left(\left[A\right]\left[B\right]-\left[A\right]\left[bA\right]\right)-\left(\left[B\right]\left[bA\right]-{\left[bA\right]}^{2}\right)$$

$$0={\left[bA\right]}^{2}-\left(\left[A\right]+\left[B\right]+K_{d}\right)\left[bA\right]+\left[A][B\right]$$

This equation has the solution

$$\left[bA\right]=\frac{\left(\left[A\right]+\left[B\right]+K_{d}\right)}{2}\pm \sqrt{\frac{{\left(\left[A\right]+\left[B\right]+K_{d}\right)}^{2}}{4}-\left[A\right]\left[B\right]}$$

where the negative root is the relevant one

$$\left[bA\right]=\frac{\left(\left[A\right]+\left[B\right]+K_{d}\right)}{2}-\sqrt{\frac{{\left(\left[A\right]+\left[B\right]+K_{d}\right)}^{2}}{4}-\left[A\right]\left[B\right]}$$

Substituting concentrations for number of antibodies/the number of molecules per mole (NA)/volume (V) yields

$$\frac{bA}{\left(N_{A}V\right)}=\frac{\left(\frac{A}{\left(N_{A}V\right)}+\frac{B}{\left(N_{A}V\right)}+K_{d}\right)}{2}-\sqrt{\frac{{\left(\frac{A}{\left(NAV\right)}+\frac{B}{\left(NAV\right)}+K_{d}\right)}^{2}}{4}-\frac{AB}{{\left(N_{A}V\right)}^{2}}}$$

or simplified

(Equation 3) $$bA=\frac{A+B+K_{d}N_{A}V}{2}-\sqrt{\frac{{\left(A+B+K_{d}N_{A}V\right)}^{2}}{4}-AB}$$

where.

bA = number of biomolecule-bound antibodies.

*A* = total number of antibodies A.

*B* = total number of target biomolecules B.

*K*_d_ = Antibody affinity (M)

*V* = the reaction volume (dm^3^)

N_A_ = Avogadro's constant (6.022 × 10^23^ molecules mole^−1^)

If the target and nontarget cells are mixed, the total number of biomolecules (*B*) will be:

$$B=\left(B_{T}C_{T}+B_{N}C_{N}\right)$$

where.

*C*_T_ = the number of target cells.

*C*_N_ = the number of nontarget cells.

*B*_T_ = the number of biomolecules on *C*_T_

*B*_N_ = the number of biomolecules on *C*_N_

The number of antibodies *A* bound to biomolecules *B* on target cells at equilibrium will be equal to the total number of bound antibodies on target and nontarget cells multiplied by the ratio between biomolecules on target cells and the total number of biomolecules (biomolecules on both target and nontarget cells):

(Equation 4) $$bA_{T}=bA\times \frac{B_{T}C_{T}}{B_{T}C_{T}+B_{N}C_{N}}$$

Furthermore, the combination of Equations 3 and 4 yields

(Equation 5) $$bA_{T}=\left(\frac{A+B+K_{d}N_{A}V}{2}-\sqrt{\frac{{\left(A+B+K_{d}N_{A}V\right)}^{2}}{4}-AB}\right)\times \left(\frac{B_{T}C_{T}}{B_{T}C_{T}+B_{N}C_{N}}\right)$$

Since not all antibodies are recovered after selection, the number of recovered antibodies (rA_T_) is given by:

(Equation6) $$rA_{T}=\left(\left(\frac{A+B+K_{d}N_{A}V}{2}-\sqrt{\frac{{\left(A+B+K_{d}N_{A}V\right)}^{2}}{4}-AB}\right)\times \left(\frac{B_{T}C_{T}}{B_{T}C_{T}+B_{N}C_{N}}\right)\right)\times E\times Y$$

where.

*E* = the fraction of antibodies eluted from target cells.

*Y* = the fraction of recovered target cells.

In the n-CoDeR library, the average copy number of each antibody is 2,000, with a 10% display level. Hence, *A* in selection 1 was estimated to be 200. In subsequent selections, *A* was calculated as the number of recovered antibodies (rA_T_) in the previous selection multiplied by the amplification factor. The amplification factor was experimentally determined from titrated phage numbers (or estimated to be 10,000, 100,000, and 10,000 between selections 1–2, 2–3, and 3–4, respectively, during selection optimization). *E* was estimated to be 0.5, and *Y* was experimentally determined (or estimated to be 0.5 during selection optimization).

In this study, we optimized the selection conditions to allow the identification of antibodies against 1) biomolecules with ≥5,000 copies per target cell and no expression on nontarget cells and 2) biomolecules upregulated at least five times (compared to nontarget cells) and expressed at ≥200,000 copies on target cells.

The experimentally observed antibody enrichment profiles were matched to *in silico*-generated enrichment profiles to group antibodies according to predicted specificities for defined categories of biomolecules. Each enrichment profile consisted of the experimentally determined, or *in silico* calculated, antibody frequencies in pools from selections 1 to 4.

For *in silico* modeled enrichment profiles, the experimental selection parameters were implemented in Equation 6 above to simulate the recovery of antibodies (rA_T_) to different biomolecule categories. The estimated frequency of a phage antibody that was selected in an antibody- and biomolecule-dependent manner in the phage pool (FA_T_) was then calculated as

(Equation 7) $$FA_{T}=\frac{rA_{T}}{\left(\sum_{1}^{n}rA_{T}\right)}\;x\;HR$$

where.

FA_T_ = frequency of recovered antibodies specific for a given type of target cell biomolecule

rA_T_ = number of recovered antibodies specific for a given type of target cell biomolecule.

$\sum_{1}^{n}rA_{T}=$ Total number of recovered antibodies specific for all types of target cell biomolecules

HR = hit rate, the fraction of phage antibodies that have been enriched in an antibody-dependent, target cell biomolecule-dependent manner. The HR was experimentally determined as described below.

$\sum_{1}^{n}rA_{T}$ equals the total number of antibodies binding to either category of target cell differentially expressed biomolecules. In this study, phage-antibody binding to biomolecules expressed throughout the experimentally determined expression range (5×10^3^ to 1×10^6^ copies/target cell) was modeled, assuming the same median affinities (K_d_ = 10 nM) and the same number of antibodies specific for different categories of biomolecules, being present in the unselected naive antibody library.

#### Definition of hypothetical biomolecules

To enable computational modeling, hypothetical biomolecules were characterized and defined based on their distinct and different (theoretical) expression, ranging from lowest to highest estimated, in target and nontarget samples. The upper range of biomolecule expression to model was estimated to be 1,000,000 copies per target cell. This upper range was determined by the number of receptors per cell targeted by the five most highly enriched antibodies during selection using fluorochrome-conjugated antibodies and quantification beads (Bang Laboratories, 815B) according to the manufacturer’s instructions. The lower range of biomolecule expression to model was estimated to be 5,000 receptors per target cell. The selections were optimized so antibodies to receptors with lower expression levels should not be retrieved and hence should not affect the enrichment profiles of other antibodies. Hypothetical biomolecules in this range (5,000–1,000,000), with expression levels increasing approximately 5-fold and comprising target sample restricted biomolecules, upregulated biomolecules, and biomolecules similarly expressed in target and nontarget samples, were defined. The numbers of (hypothesized) biomolecules in target and nontarget samples were kept constant during modeling.

#### iLaMA optimization of selection conditions

The number of target and nontarget cells needed to recover and enrich 10 nM antibodies against 1) receptors with ≥5,000 copies or more per target cell and no expression on nontarget cells and 2) receptors upregulated at least five times on target cells compared to nontarget cells and with ≥200,000 copies on target, cells were calculated using Equation 6. The total number of antibodies *A* in selection one was estimated to be 200 since the average copy number of each antibody in the n-CoDeR library is 2,000, with a 10% display level. In subsequent selections, *A* was calculated as the number of recovered antibodies (rA_T_) in the previous selection multiplied by the amplification factor. Amplification factors of 10,000, 100,000 and 10,000 between selections 1–2, 2–3 and 3–4, respectively, were used in calculations. The fraction of antibodies eluted from target cells *E* and the fraction of recovered target cells *Y* were estimated to be 0.5.

#### iLaMA-guided cell selections

For selections with nontarget cell competition, DU145 target cells were harvested, washed, biotinylated with EZ-Link Sulfo-NHS-SS-Biotin (Thermo Fisher Scientific), and labeled with anti-biotin microbeads (Miltenyi Biotec) according to the manufacturer’s instructions. Labeled target cells (10, 2.5, 5, or 5 million cells in selections 1, 2, 3, and 4, respectively) were mixed with approximately 1000 times excess of Jurkat nontarget cells and incubated with the n-CoDeR scFv phage display library^26^ (BioInvent) at +4°C overnight on a rocking platform. The cell-phage mixture was loaded on a MACS column followed by extensive washing. After washing, bound phages were eluted with trypsin, 1 mg/mL (Sigma-Aldrich) for 30 min at room temperature before inactivation with aprotinin, 0.2 mg/mL (Sigma-Aldrich). Exponentially growing *E*. *coli* HB101F′ (in-house constructed from *E*. *coli* HB101, Thermo Fisher Scientific) were infected with the eluted phages, spread on selective agar plates, and incubated overnight at 30°C. Colonies were pooled and cultivated using R408 (Agilent Technologies) as a helper phage to produce amplified phage pools for use in consecutive selections. Eluted and amplified phage pools were titrated by infection of exponentially growing *E*. *coli* HB101F′ with serial dilutions of the collections. The bacteria were spread on selective agar plates and incubated overnight at 37°C before counting the resulting colonies. The amplification factor was then calculated as the number of phages used for selection divided by the number of phages eluted in the previous selection.

In selections without nontarget cell competition, phages were incubated with 10, 2.5, 5, or 5 million DU145 cells in selections 1, 2, 3, and 4, respectively, for 4 h at +4°C on a rocking platform. Cells were washed with PBS four times before phages were recovered and processed as described above.

#### Hit rate determination

Phagemid DNA was purified (Miniprep kit, Qiagen), and genes encoding scFv were ligated into a protein expression vector (BioInvent) used to transform chemically competent *E*. *coli* Top10 (Thermo Fisher Scientific). Transformed bacteria were spread on selective agar plates and single colonies were picked and used for production of soluble scFv in 96-well microtiter plates. 300-1,100 scFv-containing supernatants from each selection were filtered (0.45 μm Millipore), and 25 μL/well was added to a 1:1 mixture of DU145 cells and CellTrace CSFE-labelled (Thermo Fisher Scientific) Jurkat cells (50,000 cells in 25 μL PBS +0.5% BSA/well) and left to bind for 1 h at +4°C. After washing, scFv binding to live cells (eBioscience Fixable Viability Dye eFluor 780, Thermo Fisher Scientific) was detected using anti-His-AF647 (R&D Systems) and analyzed by flow cytometry (iQue, Intellicyt Sartorius FortCyt v 8.0). The hit rate was determined as the fraction of analyzed clones with a mean fluorescence intensity on target cells at least three times higher than an isotype control. The analysis was performed for selection 1–4 with nontarget cell competition and selection 2–4 without nontarget cell competition. The hit rate for selection 1 without competition was estimated to be 0.09%, the same as for selection 1 with competition.

#### iLaMA calculation of predictive signatures

*In silico* predicted enrichment signatures, defined as the expected antibody frequencies observed over four consecutive selection rounds of 10 nM antibodies targeting receptors with varying expression profiles, were calculated using Equations 6 and 7. The total number of antibodies *A* in selection 1 was set to 200 since the average copy number of each antibody in the n-CoDeR library is 2,000 with a 10% display level. In subsequent selections, *A* was calculated as the number of recovered antibodies (rA_T_) in the previous selection multiplied by the amplification factor (experimentally determined). The fraction of antibodies eluted from target cells *E* was estimated to be 0.5, and the fraction of recovered target cells *Y* was experimentally determined. Phage-antibody binding to biomolecules expressed throughout the experimentally determined expression range (5×10^3^ to 4×10^6^ copies/target cell) was modeled, assuming the same median affinities (K_d_ = 10 nM) and the same number of antibodies specific for different categories of biomolecules, being present in the unselected naive antibody library.

#### Sequence library preparation and illumina sequencing

Phagemid DNA was purified from enriched phage pools using the QIAprep Spin Miniprep Kit (Qiagen). One-step PCR using *PfuUltra* II Fusion HS DNA Polymerase (Agilent) was performed to amplify scFv encoding genes from phagemid DNA and attach Illumina adaptors and indexes to the samples. The reaction volume was 50 μL/sample, with 50 ng template and 0.2 μM of each primer. Samples for MiSeq sequencing were amplified using two primer pairs: the first covering CDR-H1, CDR-H2, and CDR-H3, and the second covering CDR-L1, CDR-L2, CDR-L3, and CDR-H3. Samples for NextSeq sequencing were amplified using a primer pair that covers CDR-H3. Reverse primers include a 10-bp index sequence to facilitate multiplexing. Primer sequences are listed in the key resources table. PCR amplification was carried out with the following conditions: 95°C/2 min; 12 cycles of 95°C/20 s, 62°C/30 s, 72°C/30 s; followed by 72°C/3 min. PCR products were purified from a 2% agarose gel (MinElute Gel Extraction Kit, Qiagen), quantified (Qubit dsDNA HS Assay Kit, Thermo Fisher Scientific), and analyzed for purity and size on an Agilent 2100 Bioanalyzer using the Agilent 1000 DNA kit and Agilent 2100 Expert software (version B.02.08.SI648(SRI)). The concentration of pooled sequence libraries was measured using the KAPA Library Quant Kit Universal qPCR Mix (Roche, KK4824).

Samples for MiSeq were combined on a flow cell (Illumina MiSeq Reagent Kit v3 (600 cycles)) with 10% PhiX added and sequenced on MiSeq using paired-end reads to a median depth of 8.5 million usable reads/sample. Samples for NextSeq were combined on four flow cells (Illumina NextSeq 500/550 High Output Kit v2.5 (300 cycles)) with 25% PhiX added and sequenced on an Illumina NextSeq 500 using single reads to a median depth of 25 million usable reads/sample. Base-calling and demultiplexing were performed using bcl2fastq version 2.20.0.422, and the quality of the resulting fastq files was then inspected using fastQC version 0.11.8 with default settings. For each sequence, the number of reads was normalized to the total number of reads in the corresponding pooled library. Sequences with less than two reads were omitted from further analysis.

#### Identification of reference receptors covering the expression range of diagnostically and therapeutically relevant targets

Ten cell surface receptors with varying gene expression profiles on target cells (DU145) and nontarget cells (Jurkat) were identified through searches in the Cancer Cell Line Encyclopedia^51^ and literature^52^ (Table S2). Their cell surface expression was measured by flow cytometry using fluorochrome-labeled antibodies (key resources table) and quantification beads (Bang Laboratories, 815B) according to the manufacturer’s instructions.

#### Generation of antibodies targeting reference receptors

The unselected n-CoDeR library or amplified phages from cell selection 2 with nontarget cell competition were used for selections against the recombinant extracellular domain of the reference receptors (key resources table) using polystyrene beads (Polysciences, 17175). 4 beads/receptor was coated with 25 pmol receptor/bead at +4°C over night. The beads were washed and incubated with the phage stock at +4°C over night. After washing, binding phages were eluted and amplified as described above for cell selections. For selections starting with the n-CoDeR library, a second selection on the recombinant protein was performed, followed by a third selection on DU145 cells. Phage-bound antibodies were converted to soluble scFvs, expressed, and analyzed by flow cytometry as described for Hit-rate determination. ScFvs binding to DU145 cells were analyzed for binding to the respective receptor by ELISA. Reference receptors were coated onto plates overnight at +4°C. The next day, scFv supernatant diluted 1:4 in PBS with 0.05% Tween 20 and 0.45% fish gelatin (both from Sigma-Aldrich) was left to bind the washed ELISA plate for 1 h at room temperature. Unbound material was removed, bound scFv was detected using an AP-conjugated anti-FLAG M2 antibody (Sigma-Aldrich), followed by the addition of a luminescent substrate (CDP Star Emerald II, Thermo Fisher Scientific), and plates were read in a plate reader (Tecan Ultra with Tecan Magellan v.3.0). All receptor binding clones were cherry-picked, grown overnight in 96-well microtiter plates and Sanger sequenced.

#### Matching experimentally observed and in silico generated antibody enrichment signatures

The frequency of individual antibody clones throughout the consecutive selections (F_S1_, F_S2_, F_S3_, F_S4_) was obtained from the NextSeq data. The antibodies were classified in three steps.

First, guided by *in silico* signatures for antibodies to all categories of target biomolecules in selections with nontarget cell competition, the antibodies were classified as relevant binders if F_S2_ > F_S1_ while F_S2_ and F_S3_ > 0 (Data S1). Antibodies not meeting these criteria were classified as nonenriched.

Second, relevant binders were classified into three binder types by matching experimentally observed and *in silico* generated antibody enrichment signatures from selections with nontarget cell competition. Experimentally determined F_S4_ was compared to the predicted frequency for binders with 10 nM affinities for receptors expressed at 10^5^ and 10^6^ copies/target cell with no nontarget cell expression (1.3 ppm and 750 ppm, respectively) to guide the classification. The antibodies were classified as binding receptors with target cell expression

1) **>1,000,000** (antibodies with a frequency signature higher than the 1,000,000 predicted signature) - inclusion criteria: F_S4_ > 750 ppm
2) **100,000–1,000,000** (antibodies with a frequency signature between 100,000–1,000,000 predicted signatures) - inclusion criteria: 1.32 ppm < F_S4_ < 750 ppm
3) **<100,000, or > 5-fold upregulated receptors** (antibodies with a frequency signature lower than the 100,000 predicted signature) - inclusion criteria: F_S4_ <1.32 ppm

Third, antibodies classified as binding receptors with <100,000 copies on target cells or upregulated receptors were further classified by comparing signatures from selections with and without nontarget cell competition. The antibodies were classified as binding to

1) **Upregulated** receptors - inclusion criteria $\frac{F_{S4}\;without\;competition}{F_{S4}\;with\;competition}$ > 100

or, if F_S4_ with competition = 0; F_S4_ without competition >0 and $\frac{F_{S3}\;without\;competition}{F_{S3}\;with\;competition}$ >10

2) **Low expressed, target cell-restricted** receptors - inclusion criteria F_S1_ ≠ 0 and F_S2_, F_S3_, F_S4_ = 0 (all without nontarget cell competition)
3) **Unclassified** – not meeting any of the criteria above.

#### Production of IgG identified with predictive signatures

Complete scFv sequences needed for clone synthesis were obtained from the MiSeq data. VH and VL + CDR-H3 sequences were combined based on the CDR-H3 sequence. In cases where one CDR-H3 was associated with more than one set of VH and VL sequences, the frequencies in the two libraries were used to join the correct VH/VL pair.

Antibody genes were synthesized (Twist Bioscience) and ligated into a vector containing genes encoding the heavy and light chain constant regions of a human IgG1 antibody (BioInvent). Suspension adapted HEK 293 EBNA (ATCC, suspension adapted in-house) in Freestyle293 medium (Thermo Fisher Scientific) supplemented with 10% Pluronic F-68 (Thermo Fisher Scientific) was transiently transfected using PEI (Polyscience Inc) in 24-well plates. The cells were incubated at +37°C, 8% CO2, 300rpm for 4h before addition of UltraPepTM Soy (Sheffield Bio-Science). After another 6 days of incubation, cell supernatants were harvested and incubated with MabSelect resin (GE Healthcare) to allow antibody binding, followed by transfer of the resin to a 96-well filter plate (Thermo Fisher Scientific) for washing and elution of purified antibodies using 100mM Glycine, pH 2.8. Purified hIgG1 antibodies were dialyzed (DispoDialyser, Harvard Apparatus) to PBS buffer.

#### Confirmatory binding analysis of antibodies identified by iLaMA

Purified IgG was diluted to 100 μg/mL, titrated in 25 μL PBS +0.5% BSA, added to 50,000 DU145 (target) and Jurkat (nontarget) cells/well and left to bind for 1 h at +4°C. After washing, IgG bound to live cells was detected using an APC-conjugated anti-human-Fc antibody (Jackson Immunoresearch, 109-136-098) together with a live/dead cell marker (SYTOX green, Thermo Fisher Scientific) and analyzed by flow cytometry (iQue, Intellicyt Sartorius using FortCyt v 8.0). To generate a calibration curve to transform the MFI at saturated binding to the receptor number, a subset of IgGs with different signal intensities at saturated cell binding concentrations was selected for receptor number determination. Purified IgGs were labeled with AF647 using an Alexa Fluor 647 carboxylic acid succinimidyl ester (ThermoFisher Scientific) according to the manufacturer’s instructions. Labeled antibodies were used for receptor number determination using calibration beads (Bang Laboratories, 816) according to the manufacturer’s instructions. The assay detection limit (receptor number for isotype control) was 1,000 receptors/cell.

### Quantification and statistical analysis

Statistical analysis was performed using GraphPad Prism 9 and conducted by a Mann-Whitney test with ∗p ≤ 0.05 (Figure S4C).

## Data Availability

• Anonymized antibody frequencies following each selection can be found in Data S1. • This paper does not report original code. • Any additional information required to reanalyze the data reported in this paper is available from the lead contact upon request.

## Supplemental information

Document S1. Figures S1–S4 and Tables S1 and S2

Data S1. Antibody frequencies in the selected phage pools, related to STAR Methods

Document S2. Article plus supplemental information
