# Supplementary material for: Sequence enrichment profiles enable target-agnostic antibody generation for a broad range of antigens
Source: Cell Rep Methods. 2023 May 9;3(5):100475. doi: 10.1016/j.crmeth.2023.100475 (PMC10261905; doi:10.1016/j.crmeth.2023.100475)
Supplement: Document S1. Figures S1–S4 and Tables S1 and S2 [file mmc1.pdf]

Cell Reports Methods, Volume *3*

Supplemental information

Sequence enrichment profiles enable target-agnostic antibody generation for a broad range of antigens

Jenny Mattsson, Anne Ljungars, Anders Carlsson, Carolin Svensson, Björn Nilsson, Mats Ohlin, and Björn Frendéus

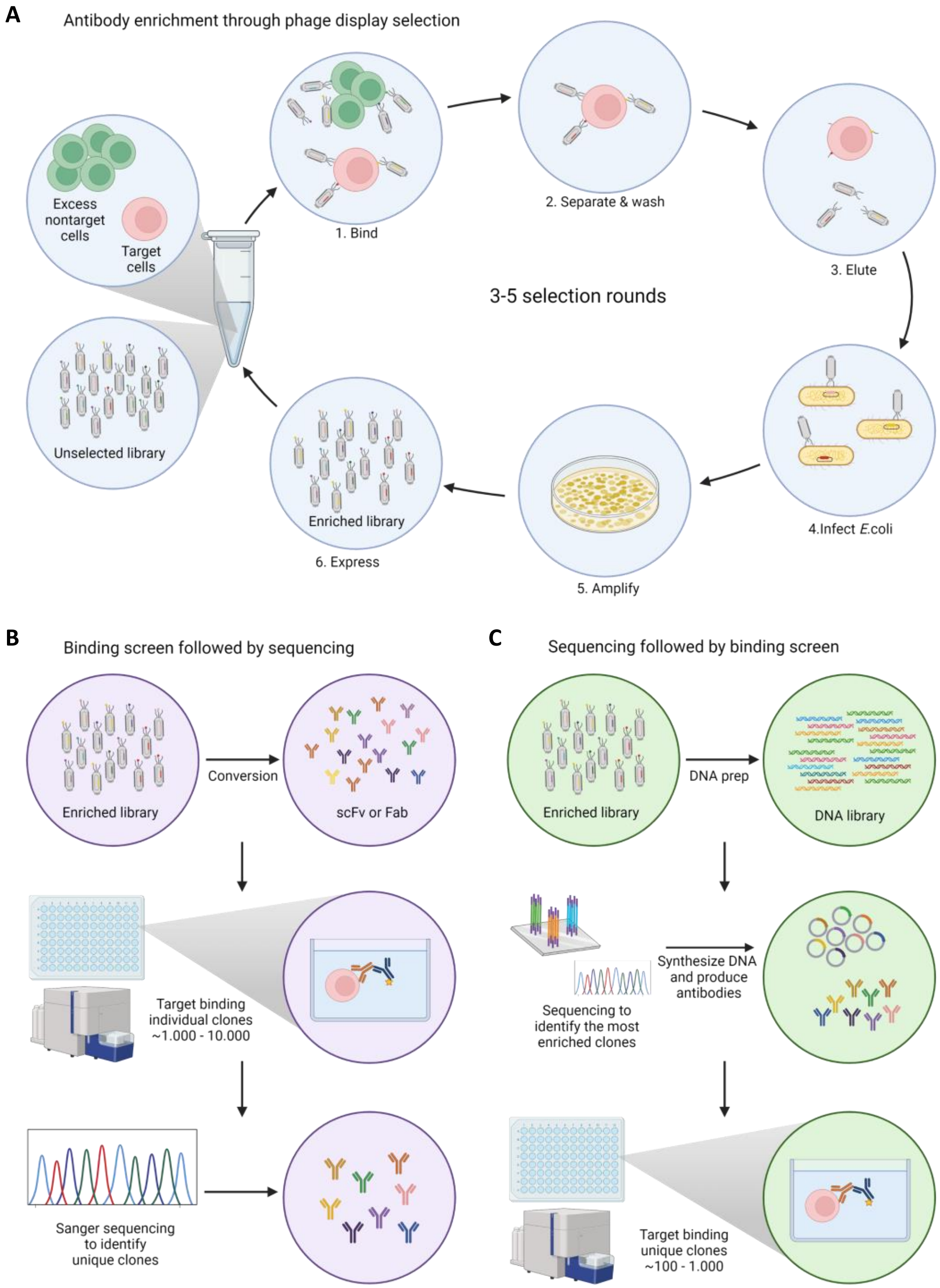

Figure S1. Current approaches to target-agnostic antibody generation. Related to Figure 1.

A. Target cells and an excess of nontarget cells are mixed with a phage display library. The displayed antibodies are allowed to bind before target cells with binding phages are separated from nontarget cell-bound phages and unbound phages. Target cell-specific phages are eluted and expanded in *E. coli* to produce an enriched library. The process is typically repeated 3-5 times in order to generate a library with sufficient enrichment of target cell-specific phage-antibodies. **B.** The enriched antibody library is converted to a soluble antibody format (scFv or Fab) and expressed as individual clones. These are screened for binding to target cells, followed by Sanger sequencing of binding clones to identify unique clones. **C.** Antibody-coding DNA from the enriched library is sequenced, either in pool by massively parallel sequencing to reveal the most enriched clones, or by Sanger sequencing of individual clones to identify unique clones. Resulting clones (the most enriched or unique) are produced and tested for binding to target cells.

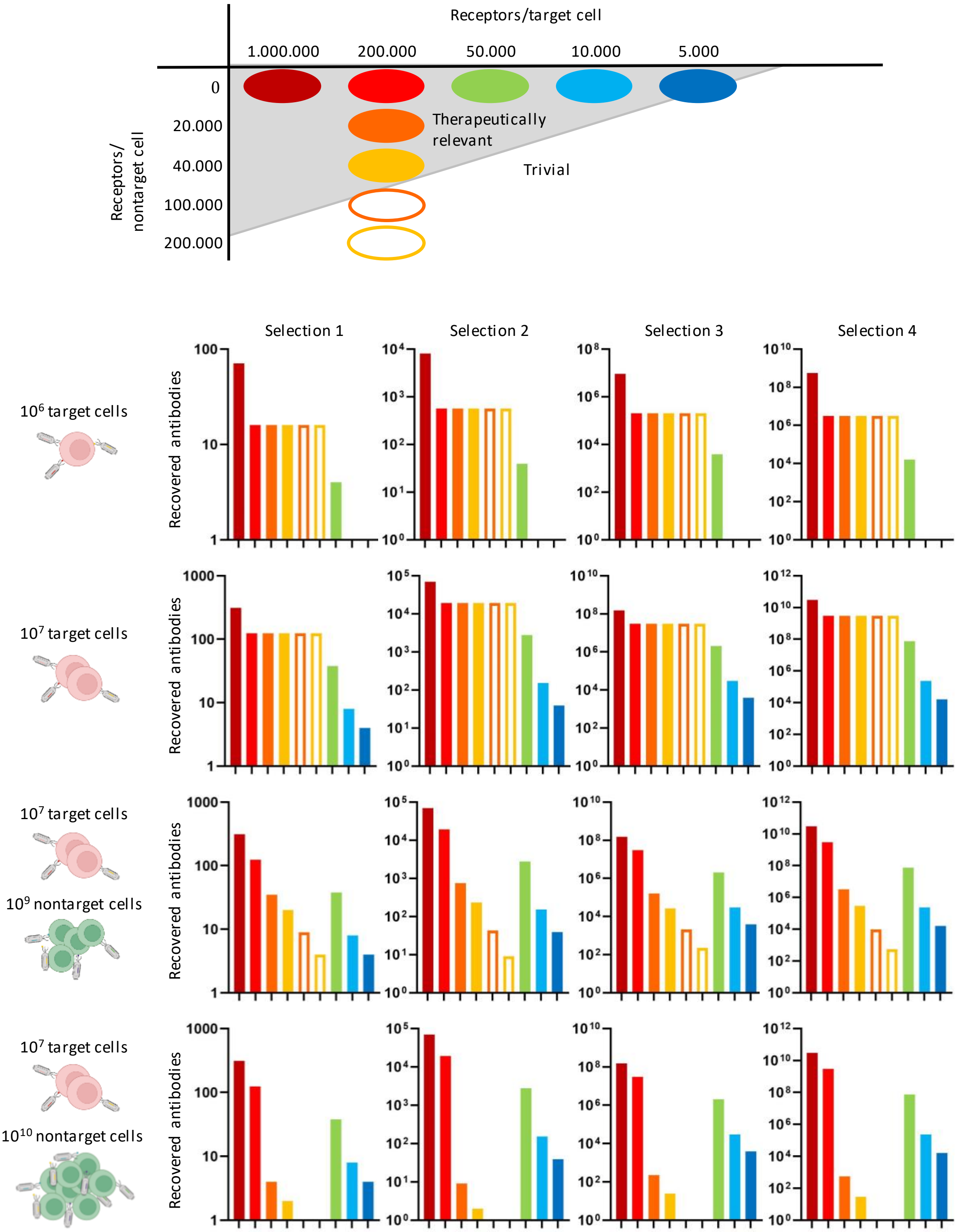

Figure S2. ***In silico*** **optimization of cell numbers for selection.** Related to Figure 2.
Calculated recovery of 10 nM binders targeting receptors expressed at 5,000, 10,000, 50,000, 200,000, or 1,000,000 receptors/target cell with no expression on nontarget cells, or receptors upregulated 10x, 5x, 2x, or 1x on target cells compared to nontarget cells using the indicated numbers of target and nontarget cells. The cell numbers used in the selections were optimized to enrich binders to receptors expressed at > 5.000 copies/target cell or upregulated > 5x on target cells, and to remove binders to receptors upregulated < 5x on target cells (lower panel).

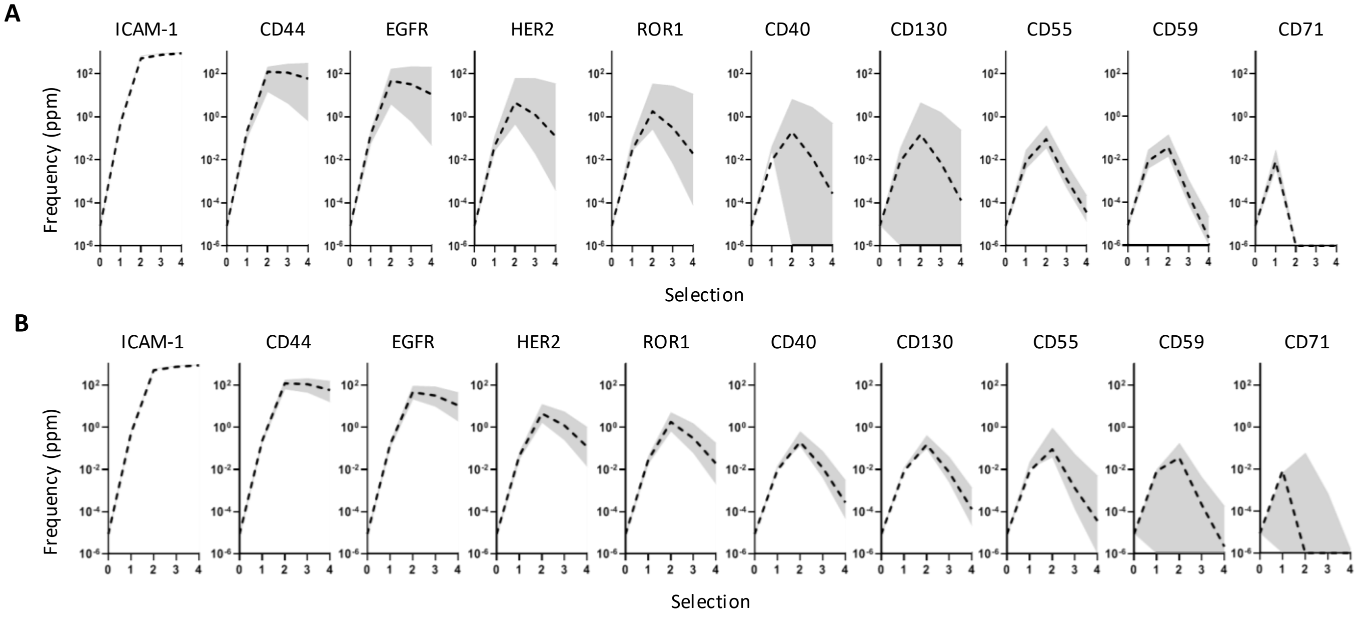

Figure S3. Variation in predicted enrichment signatures. Related to Figure 2.
In silico modeled signatures for ten reference receptors in selections with nontarget cell competition. Gray areas indicate the variation with different parameters. **A.** Variation of antibody affinity. Black dotted lines show the predicted signatures for 10 nM antibody affinity, gray areas indicate 10 times higher or lower affinity (1-100 nM). **B.** Variation of number of cell surface receptors. Black dotted lines show the predicted signatures for measured number of cell surface receptors according to supplementary table 1, gray areas indicate 2 times higher or lower receptor numbers.

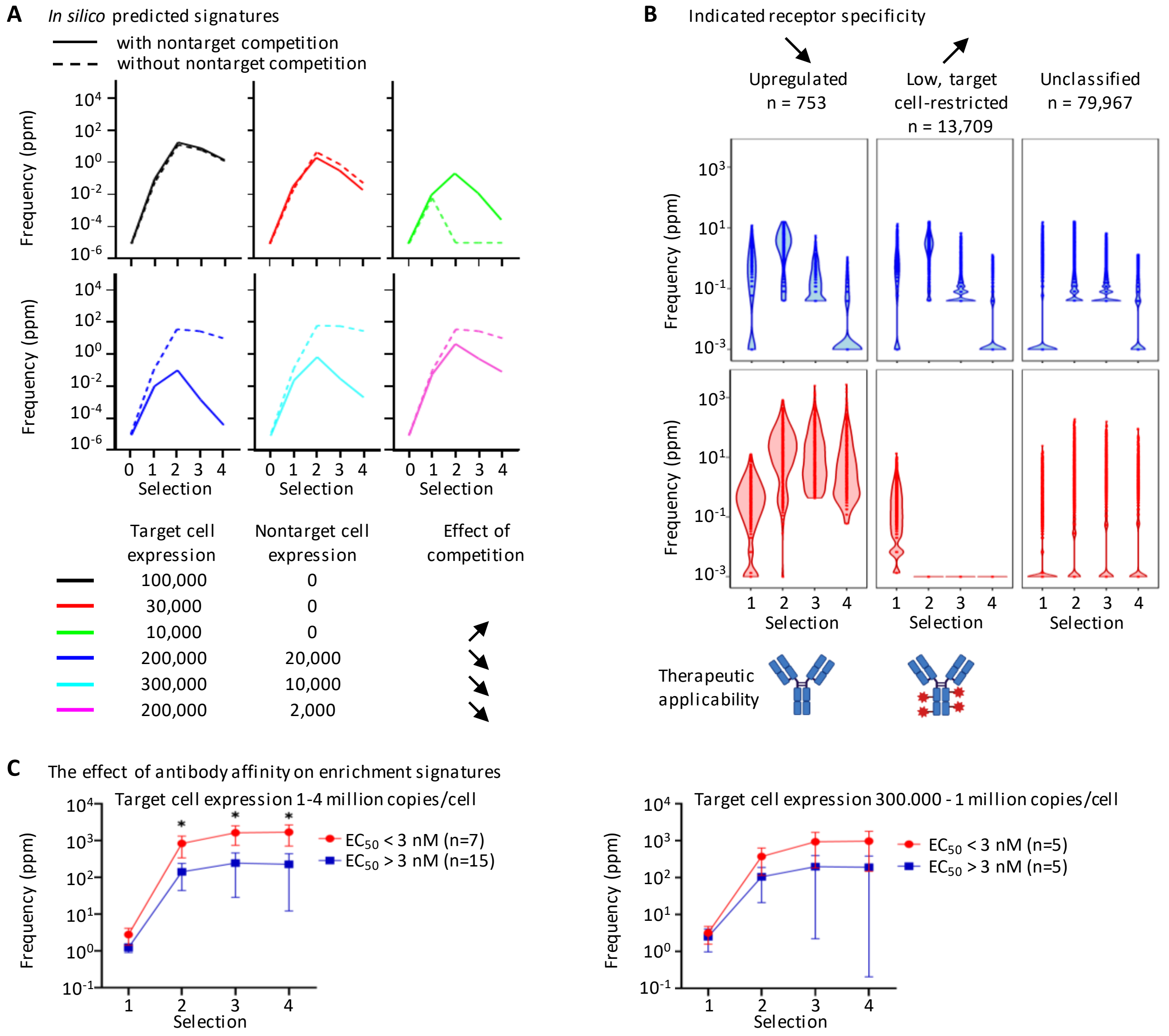

Figure S4. Antibody enrichment signatures. Related to Figure 3.

A. The low-frequency enrichment signature region (blue in Figure 3A), represents antibodies targeting receptors expressed at <100,000 copies/target cell and receptors that are upregulated on target compared to nontarget cells, as shown by *in silico*-derived enrichment signatures (solid lines). Signatures from selections without nontarget cell competition (dotted lines) indicate that these antibodies can be further classified guided by comparative analyses of signatures from selections with and without nontarget cell competition. The lower panel indicates the effect of competition on the enrichment signatures for binders to receptors with indicated expression levels. **B.** Antibodies classified as targeting receptors expressed at <100,000 copies/target cell or upregulated receptors (blue in Figure 3A), have been further classified based on signatures from selections without nontarget cell competition. Blue violins show selections with competition and red violins selections without competition. Antibodies have been classified either as binders to upregulated receptors (decreasing frequency in selections with competition), binders to low expressed, target cell-restricted receptors (increasing frequency in selections with competition) or have not been classified (not clearly affected by competition selection). **C.** Antibodies against target cell-restricted receptors with similar expression levels were divided into two groups based on their EC_{50} values in flow cytometry. The antibody frequency in the selected phage pools after selections 1-4 is plotted as the mean frequency (ppm) ± SEM. The left plot shows the frequency of antibodies targeting receptors with an expression level of 1-4 million copies/cell and the right plot shows the frequency of antibodies targeting receptors with an expression level of 300.000 - 1 million copies/cell. Antibodies with an EC_{50} value < 3 nM (red) are more frequent than antibodies with an EC_{50} value > 3 nM (blue) after selections 2, 3, and 4, but not after selection 1. The difference is significant for antibodies targeting receptors expressed at 1-4 million copies/cell (n=22). *Indicates $p < 0.05$ by Mann-Whitney test (GraphPad Prism, selection 2: p=0,046522; selection 3: p=0,026175; selection 4: p=0,033252).

Table S1. Hit rate determination. Related to STAR Methods

**Selections with nontarget cell competition**		
Selection	No of clones tested	Hit rate
1	1116	0.09%
2	558	68%
3	372	88%
4	372	94%
**Selections without nontarget cell competition**		
Selection	No of clones tested	Hit rate
2	384	76%
3	384	95%
4	384	99%

Table S2. Number of receptors expressed on target and nontarget cells, experimentally determined by flow cytometry. Related to Figure 2.

Receptor	Target cell expression	Nontarget cell expression
ICAM-1	4,700,000	BD*
CD44	400,000	BD*
EGFR	200,000	BD*
HER2	50,000	BD*
ROR1	30,000	BD*
CD40	10,000	BD*
CD130	8,000	BD*
CD55	200,000	20,000
CD71	200,000	100,000
CD59	400,000	100,000

*Below Detection limit
